# Sustainable development goal 2: Improved targets and indicators for agriculture and food security

**DOI:** 10.1007/s13280-018-1101-4

**Published:** 2018-09-28

**Authors:** Juliana Dias Bernardes Gil, Pytrik Reidsma, Ken Giller, Lindsay Todman, Andrew Whitmore, Martin van Ittersum

**Affiliations:** 1 Plant Production Systems, Wageningen University, 6700 AK Wageningen, The Netherlands; 2 School of Agriculture Policy & Development, University of Reading, Berkshire, RG6 6AH UK; 3 Sustainable Agriculture Sciences Department, Rothamsted Research, Hertfordshire, AL5 2JQ UK

**Keywords:** Country scorecard, Malnutrition, Obesity, Sustainability, Zero Hunger

## Abstract

The pursuit of global food security and agricultural sustainability, the dual aim of the second sustainable development goal (SDG-2), requires urgent and concerted action from developing and developed countries. This, in turn, depends on clear and universally applicable targets and indicators which are partially lacking. The novel and complex nature of the SDGs poses further challenges to their implementation on the ground, especially in the face of interlinkages across SDG objectives and scales. Here we review the existing SDG-2 indicators, propose improvements to facilitate their operationalization, and illustrate their practical implementation in Nigeria, Brazil and the Netherlands. This exercise provides insights into the concrete actions needed to achieve SDG-2 across contrasting development contexts and highlights the challenges of addressing the links between targets and indicators within and beyond SDG-2. Ultimately, it underscores the need for integrated policies and reveals opportunities to leverage the fulfillment of SDG-2 worldwide.

**Electronic supplementary material:**

The online version of this article (10.1007/s13280-018-1101-4) contains supplementary material, which is available to authorized users.

## Introduction

Following the millennium development goals (MDGs), the first supranational development agenda ever proposed, the international community established in 2015 a new set of aspirational goals and targets that should guide the actions of every nation in the pursuit of a better world. The sustainable development goals (SDGs) cover all social, economic, and environmental dimensions of sustainability (UN 2015). While the MDGs focused solely on developing countries, the 17 SDGs are inclusive of developed countries, since concerted action among all countries is the only way to achieve prosperity without threatening planetary boundaries (Steffen et al. 2015).

SDG-2 aims to “End hunger, achieve food security and improved nutrition and promote sustainable agriculture”. Intrinsically related to society, economy, and the environment, SDG-2 is key to the success of the entire SDG agenda (FAO 2016a). Although poor countries tend to show greater reliance on farming activities, food production and consumption is fundamental to any economy and permeates every society. Meeting SDG-2 is thus likely to invoke multiple synergies and trade-offs with other SDGs, across temporal and spatial scales, ultimately underscoring the indivisible nature of the SDG agenda.

The eradication of hunger requires SDG-2 targets and indicators aligned with the four pillars of food security: availability (having available sufficient quantities of food, whose continued production also depends on a healthy environment), access (having the economic and physical means to obtain a nutritious diet), utilization (having adequate dietary intake and the ability to absorb and use nutrients in the body), and stability (ensuring the other three pillars on a consistent basis) (FAO 2008). The triple burden of malnutrition—the coexistence of undernourishment, micronutrient deficiency, and overnutrition manifest in overweight and obesity—is a growing challenge all over the world (Gómez et al. 2013) and indicates how structural changes affected the pillars of food security. Most of these changes concern production systems, the emergence of commercial food value chains and urbanization. Altogether, they led to the substitution of more diverse, nutritious diets by greater consumption of calorie-rich staples that marked the post-Green Revolution era (Gómez and Ricketts 2013; Popkin 2014), clearly calling for systems-oriented malnutrition alleviation strategies.

SDGs have been framed in a purposefully general manner, following the idea of country-led implementation. As policies are usually implemented at the (sub-)national scale, SDGs should not be overly prescriptive but rather offer guidelines adaptable to specific contexts. Yet, despite the need for flexibility, the operationalization and monitoring of the SDGs require tangible indicators and threshold values. Clear conceptual definitions are important to ensure the fulfillment of minimum standards and the comparison across countries. Monitoring performance is not only a matter of ‘shame and blame’ to stimulate countries into action; rather, it is key to inform priority actions and channel scarce resources effectively.

A growing number of indicators have been proposed for the SDGs, including SDG-2 (Kroll 2015; SDSN 2015; Sachs et al. 2016). Despite targeting the same topics (e.g., crop yields, health, and nutrition), these proposals differ markedly regarding the number of indicators, degree of detail, target region, and priority actions. The United Nations Inter-Agency and Expert Group on SDG Indicators conducted public consultations on an official SDG indicator framework. To date, 15 indicators have been set out for SDG-2, but inconsistencies remain, data for monitoring are often unavailable and their operationalization is unclear (Hák et al. 2016). While some of these UN SDG-2 indicators present objectives, others present means to achieve them; also, some present straightforward objectives, whereas others present complex ones, conditional on a set of other actions.

We seek to contribute to the design of indicators and their implementation by reviewing the UN-proposed SDG-2 indicators and suggesting improvements wherever possible. We apply these indicators to three contrasting countries—the Netherlands, Brazil, and Nigeria—to explore various food-related challenges and illustrate the need for flexible indicators. Furthermore, case studies help contextualize the necessary level of change to achieve the SDGs.

This paper has six sections. Sections “Introduction” and “Reviewing SDG-2 targets and indicators” provide an overview of SDG-2 as recommended by the UN and proposes the revision of its indicators. Sections “Case studies” and “Applying SDG-2 indicators” analyze agriculture and food security in Nigeria, Brazil, and the Netherlands using the revised indicator set. Based on the countries’ individual performance with regards to SDG-2, Section Country priorities and policies recommends policies to increase conformity with SDG-2 targets. Finally, Section “Concluding remarks” concludes with main lessons from the case studies.

## Reviewing SDG-2 targets and indicators

SDG-2 is composed of eight targets (i.e., specific, measurable, and time-bound outcomes that directly contribute to the achievement of a goal) and 15 indicators (i.e., metrics used to measure progress towards a target, generally based on available data). The first five targets (2.1–2.5), the focus of this study, are directly related to food security and agricultural sustainability. The last three (2a–2c) are market-related measures aimed at increasing agricultural investments and reducing market restriction, distortions and volatility.

Table 1 summarizes our review of targets 2.1–2.5, highlighting whether each of their indicator is (i) conceptually clear, (ii) quantifiable, and (iii) universally relevant. We then recommend improvements ranging from minor textual changes to major content-related modifications and even their replacement. The proposal of new indicators was based on the availability of empirical data and was kept to a minimum given the already extensive list of indicators with which countries are expected to comply. Ten principles from the Sustainable Development Solutions Network for setting up a robust global monitoring indicator framework guided this exercise, including universality, simplicity, and prioritization of well-established data sources (SDSN 2015).

**Table 1:** Review of the SDG-2 targets and indicators proposed by the United Nations. More details can be found in the Electronic Supplementary Material (ESM S1)

SDG-2 targets	Original indicators (UN-IAEG-SDGs)	Conceptually clear?	Quantifiable?	Universally relevant?	Edited list of indicators
[2.1] By 2030, end hunger and ensure access by all people, in particular the poor and people in vulnerable situations, including infants, to safe, nutritious and sufficient food all year round	[2.1.1] Prevalence of undernourishment	Yes	Yes (prevalence)	Yes, particularly in poor countries	[2.1.1] Prevalence of undernourishment. [2.1.2] Per capita food supply variability index. [2.1.3] Depth of the food deficit
	[2.1.2] Prevalence of moderate or severe food insecurity in the population, based on the Food Insecurity Experience Scale (FIES)	Yes, but currently unavailable	Yes (prevalence)	Yes, particularly in poor countries	
[2.2] By 2030, end all forms of malnutrition, including achieving, by 2025, the internationally agreed targets on stunting and wasting in children under 5 years of age, and address the nutritional needs of adolescent girls, pregnant and lactating women and older persons	[2.2.1] Prevalence of stunting (height for age < -2 standard deviation from the median of the World Health Organization (WHO) Child Growth Standards) among children under 5 years of age.	Yes	Yes (prevalence)	Yes, particularly in poor countries	[2.2.1] Prevalence of stunting among children under 5 years of age. [2.2.2.a] Prevalence of wasting among children under 5. [2.2.2.b] Prevalence of underweight among children under 5. [2.2.3] Prevalence of anemia among pregnant women. [2.2.4] Average protein supply. [2.2.5] Share of average protein supply of animal origin. [2.2.6] Prevalence of obesity among adults
	[2.2.2] Prevalence of malnutrition (weight for height > + 2 or < − 2 standard deviation from the median of the WHO Child Growth Standards) among children under 5, disaggregated by type (wasting and overweight)	Yes	Yes (prevalence)	Yes, particularly in poor countries	
[2.3] By 2030, double the agricultural productivity and incomes of small-scale food producers, in particular women, indigenous peoples, family farmers, pastoralists and fishers, including through secure and equal access to land, other productive resources and inputs, knowledge, financial services, markets and opportunities for value addition and non-farm employment	[2.3.1] Volume of production per labor unit by classes of farming/pastoral/forestry enterprise size	Does *labor unit* refer to total labor (specialized + non-specialized)?	Yes (changes in volume of production or income)	Target: No (not relevant to double productivity everywhere). Indicators: Yes, but not equally important across countries	[2.3.1] Yield gap. [2.3.2] Rural poverty headcount ratio at national poverty lines. [2.3.3] Prevalence of farmers earning less than the national minimum wage
	[2.3.2] Average income of small-scale food producers, by sex and indigenous status	Does *income* refer to on-farm and/or off-farm? When is someone considered *indigenous*?			
[2.4] By 2030, ensure sustainable food production systems and implement resilient agricultural practices that increase productivity and production, that help maintain ecosystems, that strengthen capacity for adaptation to climate change, extreme weather, drought, flooding and other disasters and that progressively improve land and soil quality	[2.4.1] Percentage of agricultural area under sustainable agricultural practices	What are *sustainable practices*?	Yes (%), but vague concepts pose obstacle to quantification	Depends on the definition of “sustainable practices”	[2.4.1] Water withdrawn by agriculture as a % of total withdrawal. [2.4.2] Average water productivity in agriculture. [2.4.3] Nitrogen use efficiency. [2.4.4] Average nitrogen surplus. [2.4.5] GHG emission intensity of food production. [2.4.6] Average carbon content in the topsoil. [2.4.7] Climate change vulnerability index for food. [2.4.8] Use of pesticides per area
	[2.4.2] Percentage of agricultural households using irrigation systems compared to all agricultural households	Yes	Yes (%)	No—irrigation needs, possibilities and efficiency vary across countries	
	[2.4.3] Percentage of agricultural households using eco-friendly fertilizers compared to all agricultural households using fertilizers	What are *eco*-*friendly fertilizers*?	Yes (%), but vague concepts pose obstacle to quantification	Depends on the definition of “eco-friendly”	
[2.5] By 2020, maintain the genetic diversity of seeds, cultivated plants and farmed and domesticated animals and their related wild species, including through soundly managed and diversified seed and plant banks at the national, regional and international levels, and promote access to and fair and equitable sharing of benefits arising from the utilization of genetic resources and associated traditional knowledge, as internationally agreed	[2.5.1] Number of plant and animal genetic resources for food and agriculture secured in either medium or long-term conservation facilities	How are *genetic resources* and *medium/long*-*term* defined?	Yes (absolute number)	Yes	[2.5.1] Average number of gaps in ex situ collections of selected crop gene pools. [2.5.2] Proportion of local breeds classified as being at risk out of all breeds whose risk of extinction is known
	[2.5.2] Proportion of local breeds classified as being at risk, not-at-risk or at unknown level of risk of extinction	Yes	Yes	Yes	

Our review revealed weaknesses in the original UN indicators. First, targets and indicators do not always focus on the same groups of people. Second, although indicators were phrased quantitatively, unclear concepts hinder their quantification. Indicator 2.4.1, for example, refers to the percentage of agricultural area under sustainable practices; while *percentage* is a quantifiable metric, agreeing on what *sustainability* is, when it is achieved and what it translates into at different scales can be difficult. Third, targets 2.3 (agricultural productivity), 2.4 (sustainability of food production systems), and 2.5 (genetic diversity) are less clearly defined and not always universally relevant. Their framing could lead to a variety of interpretations due to the vagueness of terms such as “sustainable” or “fair”, as well as their lack of specificity regarding the scale of enforcement and monitoring or the boundaries of “food systems”. While the main challenge concerning targets 2.1 (hunger) and 2.2 (malnutrition) is *how* to achieve them efficiently, targets 2.3–2.5 first require the definition of *what* they consist of, even prior to answering how to operationalize them.

A major challenge when selecting indicators under a specific SDG is to capture areas of overlap with other SDGs, such as the link between agriculture, nutrition, and public health (most directly relevant to SDGs 2 and 3). Multidimensional indicators that bring these together should be prioritized. For instance, the reduced incidence of non-communicable diseases (NCDs) is a target under SDG-3 (“Good health and well-being”) but calls for agricultural policies conducive of nutritious, healthy diets.

Because of their complexity, multidimensional indicators usually rely on more detailed data and are seldom available at the national level. Indicators measuring the impact of agricultural interventions on nutrition (Herforth et al. 2016; Herforth and Ballard 2016) would be useful in this context, but were designed to be applied locally. Likewise, newer indicators attempting to explore food access and dietary consumption nationally such as the Food Insecurity Experience Scale (FIES) (Ballard et al. 2013) and the Minimum Dietary Diversity for Women (MDD-W) (FAO 2016b) are still undergoing validation.

Besides underscoring the need to invest in global databases and to conduct complementary assessments locally, data limitations elicit the need for a more holistic policy approach when implementing the SDG agenda. While recognizing the relevance of several factors to food security and the limitations of unidimensional indicators (such as anthropometric or biochemical measures of malnutrition), we base our recommendations on the understanding that the simultaneous pursuit of all indicators under SDG-2 (and other SDGs) will naturally prompt integrated solutions among health, food production, nutrition, and other fields. An analogous rationale applies to the SDG agenda, as the achievement of sustainable development relies on the simultaneous and integrated implementation of all SDGs.

### Target 2.1

Data on specific groups highlighted in the target (i.e., poor, vulnerable, infants) are limited. Although the Food Insecurity Experience Scale (FIES) has the potential to capture the complexity of actual and perceived food security, FIES data are not yet available for all countries. To overcome part of these data constraints, we propose two new indicators: 2.1.2—“Per capita food supply variability index” and 2.1.3—“Depth of the food deficit”. Both can be monitored through global, readily available databases and are better aligned with the concept of food security and its pillars.

### Target 2.2

Although the indicators under target 2.2 can be monitored through globally available databases, three problems arise. First, indicators of target 2.2. do not fully cover the groups highlighted (i.e., adolescent girls, pregnant/lactating women, and elderly). Second, the phrasing is illogical; unless a base year is determined (thus fixing the range of low heights to be avoided), the prevalence will always be the same (2.5% distribution tail, i.e., − 2 SD.) and the indicator never achieved. Third, not all malnutrition conditions defined by the World Health Organization (WHO) are captured, namely: undernutrition, i.e., wasting (low weight-for-height), stunting (low height-for-age), and underweight (low weight-for-age); micronutrient-related malnutrition, i.e., micronutrient deficiencies (a lack of important vitamins and minerals) or micronutrient excess; and overweight, obesity, and diet-related non-communicable diseases (e.g., heart disease, stroke, diabetes, and some cancers).

We suggest the amendment of the original text (no allusion to distribution curve) and four new indicators: 2.2.3 (anemia among pregnant women) covers one more group mentioned in the target; 2.2.4 (protein supply) offers a proxy for food quality in the absence of data on specific micronutrients; and 2.2.5 (share of protein supply from animal sources) and 2.2.6 (obesity) are directly linked with NCDs, an increasing concern in developed and developing countries. The new indicators cover the health-malnutrition nexus more comprehensively and are readily available through the FAO database.

### Target 2.3

The text of target 2.3, particularly with regards to doubling agricultural productivity, is not universally applicable. In some countries, the pursuit of agricultural intensification collides with the pursuit of agricultural sustainability. Notwithstanding the aspirational role of SDG targets and the importance of *secure and equal access to* inputs, knowledge, etc., such abstract concepts cannot be fully captured.

Measuring productivity on a labor basis instead of e.g., land (indicator 2.1.1) may not be adequate in some contexts. Also, it may be hard to tease out variations in agricultural output stemming from changes in labor productivity vs. other inputs (e.g., machinery). The relationship between target 2.3 (agricultural productivity) and indicator 2.1 (labor productivity) is unknown and may not be proportional, posing further obstacles to the calculation of country-specific threshold values. Finally, the proposition of a global definition for *small*-*scale* under indicator 2.1.2 may create distortions. Farmers’ income level offers little insight into their living conditions unless compared against a meaningful benchmark.

We suggest the replacement of indicator 2.3.1 by “yield gap” since the latter addresses agricultural intensification relative to a country’s potential yield on a *per land* basis, offering a benchmark for productivity. As yield gap is a complex concept that involves several factors influencing agricultural productivity (Lobell et al. 2009; van Ittersum et al. 2013), we adopt the definition used in the Global Yield Gap Atlas (GYGA 2018), based on a global protocol with local application. We also suggest the replacement of indicator 2.3.2 by two new indicators related to farmers’ income level independent of scale. The first refers to the share of the rural population below national poverty lines; the second refers to the share of farmers earning less than the national minimum wage. Country-specific reference values (i.e., poverty lines and minimum wages) account for differences in currency exchange rates and purchase power parity, allowing for international comparisons and ensuring locally meaningful results.

### Target 2.4

Sustainability has social, environmental, and economic dimensions, thus permeating every SDG and SDG-2 indicator. In this sense, indicators 2.4.2 and 2.4.3 seem embedded into indicator 2.4.1, rendering unclear why emphasis has been placed on irrigation and fertilizer use but no other equally relevant aspect of environmental sustainability such as water productivity, GHGs, and pesticide use in agriculture. The vagueness and context-dependency of the term “sustainable practices” preclude cross-country comparability. Moreover, although the concepts “sustainability” and “resilience” are intimately related (Cabell and Oelofse 2012), the second is overlooked.

The use of irrigation may be sustainable or unsustainable depending on local water availability, water productivity levels, conditions of extraction and withdrawal, criteria for disposal, etc. Thus, indicator 2.4.2 should consider the pressure that irrigation poses on the renewable water resources of each country, complementing SDG-6 (dedicated to water sustainability) and indicator 2.4.3 (which addresses agriculture-related sources of water pollution). Variations in the efficiency of different irrigation systems should also be considered at country-level whenever data are available.

Concerning indicator 2.4.3, the term “eco-friendly” is poorly defined. In some contexts, the volume and form of fertilizer application may be just as relevant for environmental conservation as the type of fertilizers (e.g., too much manure can also lead to leaching). Also, the text refers to the share of households using “eco-friendly” irrespective of their agricultural yields or total fertilizer use, which may be misleading when many small farmers use eco-friendly fertilizers but represent a small share of total food production, or when farmers use eco-friendly fertilizers but that is only a small share of their total fertilizer usage.

We suggest seven new indicators directly related to key elements of agricultural sustainability: share of water withdrawal for agriculture; average water productivity in agriculture; nitrogen use efficiency and average nitrogen surplus (Zhang et al. 2015); GHG emission intensity of food production (Carlson et al. 2016); average carbon content in the topsoil; and pesticide use per area. We also propose the adoption of the Global Adaptation Initiative (GAIN) climate change vulnerability index for food (GAIN 2015), which summarizes a country’s vulnerability to climate change in terms of food production by forecasting the evolution of key elements of food provision (see ESM S1).

### Target 2.5

A very small share of plant species is used in agriculture. Wheat, rice and maize alone provide more than half of the energy consumed by humans. This has led to a major biodiversity loss and genetic erosion. Target 2.5, aimed at the conservation of agrobiodiversity (i.e., the diversity of living organisms used in agriculture), is not only relevant for the maintenance of genetic diversity but also diet quality, resilience of production systems, and biodiversity conservation at the farm and landscape scales. Although all indicators proposed by the UN focus on important aspects of the genetic conservation in agriculture, the data to monitor indicator 2.5.1 are largely unavailable. Besides, indicator 2.5.2 could offer a distorted picture of the countries’ efforts to protect local genetic pools since the proportion of breeds cataloged in each of them varies considerably. We suggest the replacement of 2.5.1 by the average number of gaps in ex situ collections of selected crop genepools—a proxy for agricultural genetic resources secured in conservation facilities (Ramirez et al. 2009)—and the amendment of 2.5.2, so that it refers to breeds whose risk of extinction is known.

## Case studies

Nigeria, Brazil, and the Netherlands (Table 2) were selected to illustrate the operationalization of SDG-2 across different development contexts.

**Table 2:** Overview of case studies

	The Netherlands	Brazil	Nigeria
**Macro-economic factors**			
Total area (km^2^)	41 543	8 515 767	923 768
Population (2015/2016)	17 million	206 million	188 million
GDP per capita (US$, 2016)	45 210 (15th)	7495 (69th)	2640 (122nd)
HDI (0–1) (2018)	0.931 (10th)	0.759 (79th)	0.532 (157th)
**Agri-food sector**			
Employment share (%)	9% (2014)	37% (2014)	70% (2010)
GDP share (%)	9% (2016)	21% (2017)	40% (2010)
Export share (%) (2016)	21%	40%	ns
Predominant farm size	< 60 ha	~ 1000 ha	< 2 ha
Prevalent farming characteristics	Intensely managed, high yield, high application of external inputs	Intensely managed, high yield farms contrast with unproductive, low yield farms	Non-intensely managed, low yields, low application of external inputs

*HDI* Human Development Index; *ns* Statistically non-significant

### Nigeria

Agriculture is the most important non-oil economic activity in Nigeria. Most farmers operate at subsistence level, with a marketable surplus of up to 25% depending on the size of the household. Over 90% of the agricultural output is produced by small-scale farmers and low-yielding production techniques. Average maize productivity is 2 tons ha^−1^ (well below the average observed in other countries with similar climate patterns). For specific crops, the yield gap is calculated to be as high as 80% of the potential yield (World Bank 2014; GYGA 2018).

The performance of Nigeria’s agriculture is tied to macro-development issues. Problems include land fragmentation (which increases transaction costs and limits mechanization), high vulnerability to climate shocks (farmland is mostly rain-fed), and weak agricultural services (limited infrastructure, technical assistance, access to credit, and access to fertilizers) (Manyong 2005).

Costs of food imports in Nigeria have been growing at 11% per year, on average. Between 2007 and 2010, the country’s food import bill was estimated at US$628 billion (World Bank 2014). The Federal Government is under great pressure to relieve food insecurity and poverty while increasing the production of raw materials for agro-based industries through domestic production, particularly since Nigeria is projected to become the third most populous country in the world by 2050 (Van Ittersum et al. 2016).

### Brazil

Brazil is a very diverse country from the economic and agro-climatic perspectives alike. Agricultural production systems vary widely concerning scale, intensification level, and degree of diversification. According to the latest available census, family-based agriculture comprised more than 80% of the rural households but only 24% of the total agricultural area (IBGE 2006). This contrasts with the reality of three major hubs of commercial agriculture in the south-east (export-oriented crops; vertically integrated agribusiness), center-west/Mapitoba (grazing, grain and fiber production; commercial and corporate farms; large-scale, industrialized farms), and south (mostly smallholders; diversified agriculture; cooperatives and contract farming) (Chaddad 2015).

Brazil is currently the third largest exporter of agricultural goods and is projected to become the first by 2024 (OECD 2015) due to an increasing demand for food and feed, both domestically and abroad. Main products include soy, beef, coffee, sugar, oranges, ethanol, and poultry (CONAB 2014). From 2015/16 to 2025/26, official statistics predict a 30% and a 29% increase in grain and meat production, respectively (MAPA 2016). Average productivity losses due to climate change are expected to be relatively small (Assad et al. 2013). Of greater concern is the impact of Brazilian agricultural practices on the global climate and the need to invest in land use mitigation actions (La Rovere et al. 2014) such as restoration of degraded lands and farming diversification. The intensification of livestock production is deemed crucial given the link with potential land sparing, indirect land use change and associated GHG emissions (Nepstad et al. 2014).

### The Netherlands

In 2015, Dutch agricultural exports exceeded 81.6 billion euros, placing the country as the world’s second largest exporter of agricultural products (mainly horticulture and livestock) (CBS 2016; Agrimatie 2017). Dairy cattle and arable crops occupy approximately half and one quarter of the Dutch utilized agricultural area (UAA), respectively. Most agricultural systems are highly productive and intensely managed. The Dutch livestock sector is also heavily reliant on feed imports (CBS 2016).

Approximately 75% of the agricultural land is classified as high input per hectare (well-above the European Union—EU—average of 26%), being close to its economic optimal (Andersen et al. 2007). High rates of fertilizer and pesticide applications have been associated with ground and surface water contamination by nitrogen and phosphorus; although average water quality has improved over the past decade, this remains a challenge (EU 2017). Biodiversity, measured through the Farmland Birds Index, has fallen by 70% over the past 30 years (EU 2017). Other problems include soil compaction, soil contamination by heavy metals and salinization, as well as soil-borne diseases. Most GHG emissions in Dutch agriculture come from enteric fermentation and manure management (RIVM 2015). Climate change might pose risks but also have positive impacts on the yields of major crops by 2050 (Reidsma et al. 2015).

## Applying SDG-2 indicators

Table 3 shows the application of the revised indicators to each country. The UN classification of indicators per tier was adapted to indicate the availability of data and standard methodologies for their application:

**Table 3:** Calculation of revised SDG-2 targets and indicators for Nigeria, Brazil, and the Netherlands. Details on threshold values can be found in the ESM S1

Revised indicator	Tier	Data sources/calculation details	Ref year	NI	BR	NL
2.1.1—Prevalence of undernourishment (%)	I	FAO Food Security Indicators—Access—Tab “v_2.6”	2014–2016	7.0	*<* *5*	*<* 5*
2.1.2—Per capita food supply variability index (ratio of variability to distance from minimum recommended daily calorie allowance)	I	Per capita food supply variability index = *a*/(*b* − *c*), where: (a) per capita food supply variability—Stability—Tab “v_3.7” (b) per capita calorie supply—Tab “v_A.9” (c) recommended average calorie daily allowance—FAO	2011	*0.04*	*0.02*	0.03*
2.1.3—Depth of the food deficit (kcal caput^−1^ day^−1^)	I	FAO Food Security Indicators—Access—Tab “v_2.8”	2014–2016	42	*10*	8*
2.2.1—Prevalence of stunting among children under 5 years of age (%)	I	FAO Food Security Indicators—Utilization—Tab “v_4.4”	2007	**42.8**	7.1	
2.2.2.a—Prevalence of wasting among children under 5 years of age (%)	I	FAO Food Security Indicators—Tab “v_4.3”	2007	**13.4**	*1.6*	
2.2.2.b—Prevalence of underweight among children under 5 years of age (%)		FAO Food Security Indicators—Tab “v_4.5”	2007	**25.7**	*2.2*	
2.2.3—Prevalence of anemia among pregnant women (%)	I	FAO Food Security Indicators—Utilization—Tab “v_4.7”	2011	**57.9**	32.4	24.6
2.2.4—Av. protein supply (g caput^−1^ day^−1^)	I	FAO Food Security Indicators—Availability—Tab “v_1.4”	2009–2011	64	*92*	*108*
2.2.5—Share of av. protein supply of animal origin (%)	I	FAO Food Security Indicators—Availability—Tabs “v_1.4” and “v_1.5”	2009–2011	**12**	**53**	**68**
2.2.6—Prevalence of obesity among adults (%)	I	US CIA’s World Factbook (CIA 2017)	2014	*6*	18	18
2.3.1—Yield gap (%)	III	Global Yield Gap Atlas (GYGA 2018)	2004–2015	**73**	40	*20*
2.3.2—Rural poverty headcount ratio at national poverty lines (% of rural population)	I	The World Bank, Global Poverty Working Group (World Bank 2017)	2009	**52.8**	*0.0*	*0.0*
2.3.3—Prevalence of farmers earning less than the national minimum wage (%)	III	National minimum wage (ILO, 2017) Farmers’ earnings—Nigeria: NBS; Brazil: IBGE/DIEESE; Netherlands: LEI/FADN	2013	NA	**35**	*5*
2.4.1—Water withdrawn by agriculture as a percentage of total water withdrawal (%)	I	(AQUASTAT 2016) (var. 4254)	2010–2012	**44.2**	**60.0**	*0.6*
2.4.2—Av. water productivity in agriculture (kg m^−1^ year^−1^)	I	Av. water productivity in agriculture = *a*/*b*, where: (a) Total crop production (FAOSTAT 2017) (b) Total agric. water withdrawal (AQUASTAT 2016)	2010–2012	**101**	**62**	*1296*
2.4.3—Nitrogen use efficiency (kg N kgN^−1^)	III	(Zhang et al., 2015): Nitrogen Use Efficiency = NUE = *N*_yield_/*N*_input_	2011	*0.83*	0.51	**0.26†**
2.4.4—Av. nitrogen surplus (ton N km^−2^)	III	(Zhang et al. 2015): Nitrogen surplus = *N*_sur_ = *N*_input_ − *N*_yield_	2011	*0.4*	9.1	**36.9†**
2.4.5—GHG emission intensity of food production (Mg CO_2_e M kcal^−1^)	III	(Carlson et al. 2016)	2000	*0.05*	*0.08*	**0.21**
2.4.6—Av. carbon content in the topsoil (% in weight)	I	(FAOSTAT 2017) (var. 6709)	2008	**0.82**	1.21	*6.37*
2.4.7—Climate change vulnerability index for food [0–1]	III	ND-GAIN Vulnerability Index For Food (GAIN 2015)	2015	**0.706**	0.404	*0.226*
2.4.8—Use of pesticides per area (kg ha^−1^)	I	(FAOSTAT 2017) (element code: 5161)	2013	NA	*4.9*	**16.6**
2.5.1—Av. number of gaps in ex situ collections of selected crop genepools (i.e., CWR genepool gaps)	III	(Ramirez et al. 2009) and (FAO 2010)	2009	2.5	1.5	*0.0*
2.5.2—Proportion of local breeds classified as being at risk out of all breeds whose risk of extinction is known (%)	II	(FAO 2012)	2012	*0.0*	*1.2*	**35.5**

Values in bold, normal and italics indicate whether the score of a country with respect to a given indicator requires major, minor or no improvements, respectively

“NA” refers to non-available data

Empty cells refer to indicators considered non-applicable to developed countries by the FAO

*Indicates developed country’s average values

†Indicates values reported for the Netherlands by Zhang et al. (2015) which largely deviate from national estimates, as discussed in the ESM S1

- Tier I: “Methodology established and data widely available”;
- Tier II: “Methodology established but data not easily available for all countries”;
- Tier III: “Methodology and/or global data proposed through alternative sources, i.e., peer-reviewed studies and non-UN related initiatives which have proposed a methodology and possibly provided global data for a given indicator”.

All indicators can be monitored through globally available datasets except for indicator 2.3.2 (smallholders’ income), which requires country-level data. Scores obtained by Nigeria, Brazil, and the Netherlands have been coded to indicate priority areas. Italics indicate satisfactory performance, while normal and bold indicate the need for minor and major improvements, respectively. The coding reflects threshold values based on expert opinion, literature review, and/or existing classifications. The ESM S1 contains details on sources, scores and, when available, trends over time.

Overall, Nigeria performs poorly with regards to targets 2.1–2.3 and worsening trends have been observed since 2011 (see ESM S1). Despite the lack of data on the prevalence of farmers earning less than the minimum wage (indicator 2.3.3), the number of people below the poverty line (indicator 2.3.2) leaves no doubt about the need to improve Nigerian farmers’ income. The indicators associated with these targets are largely interdependent. Reducing the yield gap (Target 2.3) could play a significant role in alleviating food insecurity, both through increased domestic supply and export revenue. Agriculture represents almost half of the total withdrawal (indicator 2.4.1), yet water productivity is very low (indicator 2.4.2). Other points for improvement are soil quality (proxied by soil organic carbon), agrobiodiversity conservation and lack of resilience to climate change. Although a high NUE value indicates a low risk of nutrient loss, there is a risk of soil mining; increased fertilizer application could reduce this risk and increase productivity.

In Brazil, food security has improved substantially over the past decade, but nutrition indicators still deserve attention. Improving water productivity and nutrient use efficiency, two interlinked indicators where the country scores particularly poorly, could help reduce the yield gap. In many regions where rainfall is abundant, better management practices and smart nutrient application are crucial. Soil quality is satisfactory on average, but severe land degradation present in parts of the country—often associated with deforestation followed by the abandonment of pasturelands—should be addressed. Also, several farmers earn less than the national minimum wage (especially smallholders).

The Netherlands performs well in nutrition except for animal-based protein consumption and obesity. Despite discrepancies between numbers reported by Zhang et al. (2015) and national statistics (CBS 2016) (see ESM S1) and improvements over the past decades (CBS 2016), Dutch agriculture must still improve its nutrient use efficiency and GHG emissions—both related to high management intensity and livestock density. The poor performance of the Netherlands at indicator 2.5.2 (species at risk) contrasts with its performance at indicators 2.5.1 (ex situ conservation of genetic diversity), probably indicating that the latter is negatively biased given their better biodiversity reporting compared to Brazil and Nigeria; yet, it is known that the occurrence of bird species has declined over the past decades (Brink 2015). Finally, the country’s score for average carbon content in the topsoil (indicator 2.4.6) is disproportionately high due to the occurrence of peat soils.

Although the scores obtained by the three countries for obesity (indicator 2.2.6) do not seem alarming, trends are rapidly deteriorating in all of them. The prevalence of obesity in the Netherlands and in Europe is almost as high as in Brazil and other middle-income countries from Latin America. Prevalence in Africa and particularly Nigeria is still comparatively low, but numbers have almost doubled since 1980 (Gómez et al. 2013).

Despite uncertainties on the quality of global databases, the observations above corroborate the countries’ description in Section “Case studies” and highlight differences in their priority areas. In Nigeria, where most farms operate under sub-optimal socioeconomic conditions and resources are underutilized, agricultural production must be intensified in a sustainable manner. Reducing the yield gap could immediately alleviate the pressure for food imports and improve access to (cheaper) food. In the Netherlands, where farms are already intensely managed and current production levels are close to their agronomic potential, emphasis should be placed on making existing agricultural systems more sustainable. In fact, most pressing environmental problems stem from the high intensity of current agricultural operations in the country. In Brazil, where highly productive farms contrast with low stocking rates and production scales vary widely, intensification and greater sustainability should be pursued in parallel.

## Country priorities and policies

Agricultural practices based on the specificities of each country and their priority areas should be fostered, especially those contributing to multiple sustainability targets at once. Although it is hard to measure the exact contribution of interventions targeted at rural development, agriculture, land use and environment towards each specific SDG-2 target, some existing policies can certainly help achieve them.

The Nigerian “Agricultural Promotion Policy—2016–2020” focuses on ensuring food security through reducing food imports. It covers, among others, institutional reforms and incentives to technological development at the local level. The Empowering Novel Agribusiness-Led Employment Program mobilizes finance for youth-led agribusiness development. The Agricultural Credit Guarantee Scheme Act from 2016 offers incentives to farmers and other professionals throughout the entire agricultural supply chains. Finally, the “Green Alternative: The Agriculture Promotion Policy” launched in mid-2016 tries to boost soybean and cowpea production, chosen for their nutritional value and export potential.

In Brazil, the so-called ABC Plan was established by the Federal Government as part of the country’s National Policy for Climate Change to restore degraded lands through the dissemination of low-carbon agricultural practices. The plan encompasses investments in research and training as well as the provision of credit lines for specific practices. Payment for environmental service schemes related to agriculture such as *Produtor de Agua* and *ICMS Ecologico* deserve to be highlighted (Richards et al. 2017). Anti-deforestation plans (PPCerrado and PPCDAM), the adoption of the Rural Environmental Registry (CAR), the creation of protected areas, and voluntary market mechanisms to incentivize environmental protection (e.g., Soy Moratorium) have helped decouple agricultural expansion and deforestation (Nepstad et al. 2014). The Brazilian Program “Zero Hunger”, based on conditional cash transfers, as well as support given to family agriculture were key to leverage people above the poverty line and ensure food security (Rocha 2009).

Dutch agriculture is regulated by several EU directives on nitrate pollution, water use and biodiversity protection. Since the 2013 reforms of the Common Agricultural Policy, about 30% of the direct payments given to European farmers are linked to sustainability practices—particularly concerned with soil quality, biodiversity and carbon sequestration (Westhoek et al. 2014)—although the effectiveness of specific measures has raised debate (Pe’er et al. 2014). Measures aimed at agricultural sustainability include market mechanisms (e.g., higher standards for production, consumption and imports), waste reduction and incentives to the adoption of organic production. According to the Dutch 2014–2020 Rural Development Program, support will be directed at improving landscapes, stimulating biodiversity, and improving soil and water management in farmland. The program also includes incentives to young farmers and innovations, e.g., phosphorus recycling, urban agriculture, and biodigestors (EU 2017).

The priority areas highlighted in Section “Applying SDG-2 indicators” may require targeted action. In Nigeria, this includes high-yielding seeds, adequate rates of fertilizer, efficient irrigation, and elimination of slash-and-burn; in Brazil, practices aimed at restoring degraded lands (e.g., soil improvement through precision agriculture, minimum-tillage), enhancing on-farm diversity (e.g., crop-livestock rotation), and increasing yields in livestock systems (e.g., use of paddocks, improved grass management); in the Netherlands, reduced nitrogen/phosphorus emissions and pesticide application rates, biodiversity protection, and GHG emissions reduction. Indicators which do not appear as bold in Table 3 may present worsening trends, thus also requiring attention (such as obesity rates in the Netherlands).

## Concluding remarks

As it currently stands, the UN’s set of indicators related to SDG-2 is not universally applicable and their operationalization still requires fine-tuning. We propose a revised set of indicators that, in our view, reflects the targets under SDG-2 more comprehensively, can be readily monitored through empirical databases, is applicable to varied development contexts and allows cross-country comparison.

The newly proposed indicators are still aligned with the core idea behind each target, and their achievement would signal that the four dimensions of food security are in place. Targets 2.1 and 2.2 reflect the ability to turn food into nutrition and health, thus being directly related to Utilization. Target 2.3 is in line with Access, especially regarding people’s ability to produce and/or purchase food. Availability relates to target 2.3 with respect to yield levels as well as targets 2.4 and 2.5 (both reflective of the capacity to produce food over time depends on ecological equilibrium). Finally, Stability ensures the constant achievement of all indicators over time.

Recent structural changes in food systems have significantly affected global nutrition (Caballero 2002; Gillespie and van den Bold 2017) and must be considered in the context of the SDG-2 when designing food systems-based strategies to fight hunger and malnutrition. It is important to think of agricultural transformation pathways compatible with a more systemic thinking, where food systems contribute to food security through e.g., food production for own consumption, incentives for greater food availability, higher incomes and lower prices, gender-specific time allocation, as well as changes in consumer behavior (Gómez et al. 2013).

Yet, no set of indicators can fully capture the link between agricultural interventions, dietary change, and nutrition, which involves several complex factors within and beyond SDG-2 [e.g., food production, diet diversification, biofortification, food safety, gender empowerment, value chains, policy support, etc. (CGIAR 2014)]. Notwithstanding the obvious need for greater collaboration between the public health and agro-food scientific communities, it is only through the combined achievement of SDGs and integrated policies that sustainable food security can be met. SDGs must be considered as a single development agenda that calls for a comprehensive and integrated policy framework (Le Blanc 2015; von Stechow et al. 2016).

Proposing meaningful indicators and monitoring countries’ progress are both conditional on information availability and quality. As our analysis shows, it is paramount to invest in better global databases (Alkire and Samman 2014). When implementing the SDGs, it is important to check the reliability of existing global figures and complement them with country-level data whenever possible. A great diversity may exist at the sub-national level, making it necessary to use more detailed local information.

Not all targets have the same degree of priority in different countries, including the three case studies assessed in this paper. Even when targets imply absolute sustainability—a debatable concept, aligned with the aspirational nature of the SDGs—the discussion of national thresholds and realistic policy targets is a necessary step towards their implementation. SDG-2 indicators should be prescriptive enough to ensure comparability across countries and the adoption of minimal standards worldwide, and flexible enough to account for country’s specific challenges and demands. Finding the perfect balance requires a participatory approach and should take advantage of existing sustainability frameworks (Slätmo et al. 2016). Besides, SDG targets and indicators must be adjusted over time to accommodate socio-economic and institutional changes. Often, the development of effective policies entails multiple iterations between theory and practice.

Given the above, this paper should be seen as one step further in the process of indicator design which may inspire the formal SDG review process. The novelty and complexity of the SDG framework opens a wide range of research avenues, of which we highlight three. First, the threshold values proposed here do not say much on whether countries are on the right track to achieve SDG-2 targets, unless monitored over a longer period. Although SDG targets are the same for all countries, the pathways they will follow will differ. Historical trends may offer hints on a country’s future, but there is no guarantee that past trends will persist. Second, synergies and trade-offs across SDGs and analytical scales should be examined to inform coherent policy design under different scenarios. Third, agricultural trade among the countries examined in this paper deserves greater attention and has direct implications for nutrition and environmental indicators, especially when food access and availability are considered over the short and long terms. In all cases, the identification of research methodologies well-suited to tackle the spatial and temporal scalar complexity of sustainability targets is crucial.

## Electronic supplementary material

Below is the link to the electronic supplementary material.

Supplementary material 1 (PDF 586 kb)
