# Supplementary material for: Sustainable development goal 2: Improved targets and indicators for agriculture and food security
Source: Ambio. 2018 Sep 28;48(7):685–98. doi: 10.1007/s13280-018-1101-4 (PMC6509081; doi:10.1007/s13280-018-1101-4)
Supplement: Supplementary file 1 — Supplementary material 1 (PDF 586 kb) [file 13280_2018_1101_MOESM1_ESM.pdf]

Ambio

Electronic Supplementary Material

Sustainable Development Goal 2: improved targets and indicators for agriculture and food security

Juliana D. B. Gil, Pytrik Reidsma, Ken Giller, Lindsay Todman, Andrew Whitmore, Martin van Ittersum

This supplementary material provides:

- Greater detail on each of the SDG-2 indicators proposed (incl. justification, definition, calculation and associated sources/databases);
- Threshold values underlying the scorecard presented in Table 3 of the manuscript (accompanied by respective sources, when available); and
- Historical trends of countries' performance with respect to specific indicators, when available.

The coding system used in Table 3 of the manuscript to indicate indicator-specific threshold values is as follows:

- *Italics*: Satisfactory
- Normal: Need for minor improvements
- **Bold**: Need for major improvements

Contents:

Target 2.1 2

Target 2.2 3

Target 2.3 7

Target 2.4 9

Target 2.5 14

Historical trends for specific indicators 16

Target 2.1. 16

Target 2.2. 17

Target 2.1

Indicator 2.1.1. “Prevalence of undernourishment” (%)

- Justification: This is the traditional FAO hunger indicator, adopted as official Millennium Development Goal indicator for Goal 1 (Target 1.9). It is directly related to the achievement of food security.
- Definition according to the FAO metadata: “The prevalence of undernourishment expresses the probability that a randomly selected individual from the population consumes insufficient calories to cover her/his energy requirement for an active and healthy life. The indicator is computed by comparing a probability distribution of habitual daily dietary energy consumption with a threshold level called the minimum dietary energy requirement. Both are based on the notion of an average individual in the reference population. The indicator is calculated in three-year averages, from 1990-92 to 2014-16, to reduce the impact of possible errors in estimated Dietary Energy Supply (DES), due to the difficulties in properly accounting of stock variations in major food.”
- Source: FAO Food Security Indicators[1] - Access - Tab "v_2.6"
- Threshold values set by the FAO/WHO:
    - *Satisfactory*: ≤ 5% (average amongst developed countries in 2014-16)
    - <u>Need for minor improvements</u>: 5-10.8%
    - **Need for major improvements**: ≥ 10.8% (above world average in 2014-16)

Indicator 2.1.2. “Per capita food supply variability index” (ratio of variability to distance from minimum recommended daily calorie allowance).

- Justification: Per capita food supply variability corresponds to the variability of the "food supply in kcal caput^{-1} day^{-1}" as disseminated in FAOSTAT. It compares the variations of the food supply across countries and time. Currently, data is available for the period 1990-2013. In our paper, we combined it with average food supply to create an index that expresses *variability* while taking into account each country’s susceptibility to falling below the average calorie daily allowance recommended by the FAO.
- Calculation:
    - Per capita food supply variability index = a/(b-c), where:
        (a) per capita food supply variability - Stability - Tab "v_3.7"
        (b) per capita calorie supply – Tab “v_A.9”
        (c) recommended average calorie daily allowance by FAO (i.e. 2250 kcal day^{-1})
- Source: FAO Food Security Indicators (specific tabs are indicated above).
- Threshold values proposed:
    - *Satisfactory*: ≤ 0.10
    - <u>Need for minor improvements</u>: 0.10–0.40
    - **Need for major improvements**: ≥ 0.40

[1] The FAO Food Security Indicators are available at http://www.fao.org/economic/ess/ess-fs/ess-fadata/en/#.WRGD4tJ96Uk.

Indicator 2.1.3. “Depth of the food deficit” (kcal $caput^{-1}$ day^{-1})

- Justification: The depth of the food deficit is an important indicator of how many calories would be needed to lift the undernourished from their status, everything else being constant. It directly complements indicator 2.1.1 (i.e. “Prevalence of undernourishment”).
- Definition according to the FAO metadata: The average intensity of food deprivation of the undernourished, estimated as the difference between the average dietary energy requirement and the average dietary energy consumption of the undernourished population (food-deprived), is multiplied by the number of undernourished to provide an estimate of the total food deficit in the country, which is then normalized by the total population. The indicator is calculated in three-year averages, from 1990-92 to 2014-16, to reduce the impact of possible errors in estimated DES, due to the difficulties in properly accounting of stock variations in major food. Aggregate values are computed using a weighted population average.
- Source: FAO Food Security Indicators – Access - Tab "v_2.8"
- Threshold values proposed:
    - *Satisfactory*: ≤ 20
    - Need for minor improvements: 20-81
    - **Need for major improvements**: ≥ 81 (i.e. world average)

Target 2.2

Indicator 2.2.1. “Prevalence of stunting among children under 5 years of age” (%).

- Justification (FAO): “Child growth is the most widely used indicator of nutritional status in a community and is internationally recognized as an important public-health indicator for monitoring health in populations.”
- Definition according to the FAO metadata: “This indicator belongs to a set of indicators whose purpose is to measure nutritional imbalance and malnutrition resulting in undernutrition (assessed by underweight, stunting and wasting) and overweight.”
- Source: FAO Food Security Indicators – Utilization - Tab "v_4.4".
- Threshold values set by the FAO/WHO:
    - *Satisfactory*: ≤ 5%
    - Need for minor improvements: 5-10%
    - **Need for major improvements**: ≥ 10%

Indicator 2.2.2.a. “Prevalence of wasting among children under 5” (%)

- Justification: Wasting is a crucial indicator of malnutrition among children, being directly related to the achievement of food security.
- Definition according to the FAO metadata: Proportion of children under five whose weight for height is more than two standard deviations below the median for the international reference population ages 0-59. This indicator belongs to a set of indicators whose purpose is to measure nutritional imbalance and malnutrition resulting in undernutrition (assessed by underweight, stunting and wasting) and overweight. Child growth is the most widely used indicator of nutritional status in a community and is internationally recognized as an important public-health

indicator for monitoring health in populations. In addition, children who suffer from growth retardation as a result of poor diets and/or recurrent infections tend to have a greater risk of suffering illness and death.

- Source: FAO Food Security Indicators - Tab "v_4.3"
- Threshold values based on WHO recommendations[2]:
  - *Satisfactory*: ≤ 5% (i.e. “low” according to WHO)
  - Need for minor improvements: 5-9% (i.e. “medium” according to WHO)
  - **Need for major improvements**: ≥ 10% (i.e. “high” or “very high” according to WHO)

Indicator 2.2.2.b. “Prevalence of underweight among children under 5” (%)

- Justification: Underweight is a crucial indicator of malnutrition among children, being directly related to the achievement of food security.
- Definition according to the FAO metadata: Weight-for-age less than -2 standard deviations of the WHO Child Growth Standards median among children aged 0-5 years. This indicator belongs to a set of indicators whose purpose is to measure nutritional imbalance and malnutrition resulting in undernutrition (assessed by underweight, stunting and wasting) and overweight. Child growth is the most widely used indicator of nutritional status in a community and is internationally recognized as an important public-health indicator for monitoring health in populations. In addition, children who suffer from growth retardation as a result of poor diets and/or recurrent infections tend to have a greater risk of suffering illness and death.
- Source: FAO Food Security Indicators - Tab "v_4.5"
- Threshold values based on WHO recommendations[3]:
  - *Satisfactory*: ≤ 10% (i.e. “low” according to WHO)
  - Need for minor improvements: 10-19% (i.e. “medium” according to WHO)
  - **Need for major improvements**: ≥ 20% (i.e. “high” or “very high” according to WHO)

Indicator 2.2.3. “Prevalence of anemia among pregnant women” (%)

- Justification (FAO): “The prevalence of anemia is an important health indicator. When used with other measurements of iron status, the hemoglobin concentration can provide information about the severity of iron deficiency.”
- Definition according to the FAO metadata: “Anemia is a condition in which the number of red blood cells (and consequently their oxygen-carrying capacity) is insufficient to meet the body’s physiologic needs. (…) Iron deficiency is thought to be the most common cause of anemia globally, but other nutritional deficiencies (including folate, vitamin B12 and vitamin A), acute and chronic inflammation, parasitic infections, and inherited or acquired disorders that affect hemoglobin synthesis, red blood cell production or red blood cell survival, can all cause anemia.”
- Source: FAO Food Security Indicators – Utilization - Tab "v_4.7"
- Calculation: "Prevalence of anemia in pregnant women is the percentage of pregnant women whose hemoglobin level is smaller than 110 grams per liter at sea level. Anemia is a condition in

[2] World Health Organization. Global Database on Children Growth and Malnutrition. Available at: http://www.who.int/nutgrowthdb/about/introduction/en/index5.html
[3] Idem.

which the number of red blood cells or their oxygen-carrying capacity is insufficient to meet physiologic needs, which vary by age, sex, altitude, smoking status, and pregnancy status. In its severe form, it is associated with fatigue, weakness, dizziness, and drowsiness. Children under age 5 and pregnant women have the highest risk for anemia. The cut-off values for public health significance is 40%. A prevalence of anemia equal or higher than this level signals a severe public health problem."

- Threshold values based on FAO recommendation (above):
    - *Satisfactory*: ≤ 20%
    - Need for minor improvements: 20-40%
    - **Need for major improvements**: ≥ 40%

Indicator 2.2.4. "Average protein supply" (g $caput^{-1}$ day^{-1})

- Justification: Protein is critical to human health. An estimated 2 billion people suffer from undernutrition – a lack of access to key micronutrients – resulting in major health risks. This indicator provides information on the quality of the diet, thus complementing other indicators concerned with the number of calories ingested by humans per day.
- Definition/calculation according to the FAO metadata: The indicator is calculated in three-year averages, from 1990-92 to 2011-13, to reduce the impact of possible errors in estimated DES, due to the difficulties in properly accounting of stock variations in major food.
- Source: FAO Food Security Indicators – Availability - Tab "v_1.4"
- Threshold values based on the FAO's recommended protein intake level (~50g $caput^{-1}$ day^{-1} for a 75kg person, or 0.626 g protein kg^{-1} day^{-1})[4]:
    - *Satisfactory*: ≥ 80 g $caput^{-1}$ day^{-1}
    - Need for minor improvements: 50-80 g $caput^{-1}$ day^{-1}
    - **Need for major improvements**: ≤ 50 g $caput^{-1}$ day^{-1}

Indicator 2.2.5. "Share of average protein supply of animal origin" (%)

- Justification: Livestock products not only provide high-value protein but are also important sources of a wide range of essential micronutrients such as iron, zinc and vitamin A. However, excessive consumption of animal products can lead to excessive intakes of fat, increase the incidence of non-communicable diseases and likely have an undesirable impact on the environment.
- Calculation:
    - Share of average protein supply of animal origin = a/b, where:
      (a) average protein supply of animal origin (g/person/day) – Availability - Tab "v_1.5"
      (b) average protein supply (g/person/day) – Availability - Tab "v_1.4" (target 2.2.4)
- Threshold values based on expert opinion:
    - *Satisfactory*: 25-35%
    - Need for minor improvements: 15-25% or 35-45%
    - **Need for major improvements**: ≤15% or ≥45%

---

[4] Available at: http://www.fao.org/docrep/003/AA040E/AA040E06.htm#ch6 (see table 16).

- Notes:
    - The proposed threshold values are based on recent research indicating that the daily recommended intake of protein from animal sources is about one third of the total daily protein intake (estimated to be around 57g per person when specific population groups such as pregnant woman are considered).
    - This share of one third is also in line with optimal values from an environmental point of view. Research suggests that eating 7-27g of protein (i.e. 12-47% of 57g $caput^{-1}$ day^{-1}) is optimal from a resource use efficiency. This, however, implies feeding leftovers (e.g. co-products, food waste) and grass resources to livestock only[5].
    - This indicator must be applied and interpreted carefully. The optimal level of animal protein consumption, both from a nutritional and an environmental perspective, may differ across agricultural contexts. The former is especially where livestock is a major accessible source of protein. Also, a balanced diet can be achieved through different combinations of animal-based and plant-based protein, especially considering different plant and animal sources. We stress the need for the collection of empirical data on the intake of fruits, vegetables, fibers, processed foods, alcohol and other food items at the national level, which could offer a more complete picture of a country's risk of malnutrition.

Indicator 2.2.6. "Prevalence of obesity among adults" (%)

- Justification: Obesity rates are rapidly increasing in developed and developing countries alike, particularly in urban settings. This trend is affected by many factors including food choices (particularly increased consumption of high-fat, high-sugar and processed foods), sedentary lifestyles, genetics and cultural beliefs. Obesity is associated with a series of infectious and chronic diseases.
- Calculation: Age standardized estimates of prevalence of obesity among adults (18+ years of age). For adults, "obese" means a body mass index (BMI) greater than 30 kg m^{-2}.
    - Note: Although this is not the case in the three countries considered in our studies, alternative definitions and threshold values may be more appropriate in different ethnic populations.
- Sources: US CIA's World Factbook (https://www.cia.gov/library/publications/the-world-factbook/rankorder/2228rank.html).
    - Note: The numbers of the CIA database were double-checked with those from the latest national surveys available through the World Obesity Federation (www.worldobesity.org):
        - Nigeria (1991-94): *WHO Infobase. Richard Cooper. Department of Preventive Medicine, Loyola University Medical School 2160 S. First Ave, Maywood, IL 60153*
        - Brazil (2008-09): *Ministério da Saúde. Pesquisa de Orcamentos Familiares 2008-9. Antropometria e Estado Nutricional de Crianças, Adolescentes e Adultos no Brasil. Instituto Brasileiro de Geografia e Estatística (IBGE) 2010.*

---

[5] Van Zanten H. Feed sources for livestock: recycling towards a green planet. *Department of Animal Sciences* 2016. PhD thesis, 251pp. Available at: http://library.wur.nl/.../504683.

        - Netherlands (1998-02): *Visscher TLS, Viet AL, Kroesbergen HT and Seidell JC. (2006). Underreporting of BMI in adults and its effect on obesity prevalence estimations in the period 1998 to 2002. Obesity, 14(11): 2054 - 2063.*
- Threshold values proposed based on the WHO World Health Observatory[6]:
    - *Satisfactory*: ≤ 10%
    - Need for minor improvements: 10-20%
    - **Need for major improvements**: ≥ 20%

Target 2.3

Indicator 2.3.1. "Yield gap" (%)

- Justification: Yield gap estimates highlight where there is potential for agricultural production improvement and help identify major causes of rural poverty linked to geographic elements.
- Source: Global Yield Gap Atlas (GYGA) - www.yieldgap.org
- Definition/calculation: Yield gap (Yg) is calculated as the difference between actual yield (Ya) and potential yield (Yp) for irrigated crops, or water-limited yield (Yw) for rainfed crops. We accounted for the crops available on GYGA, where Yp and Yw are simulated considering optimal agronomic management as input (i.e. cultivar maturity, sowing date and planting density) based on dominant practices currently used by farmers.
- Note: FAO's GAEZ project[7] also calculates country-specific yield/production gap values. They were estimated by comparing potential attainable yields and production (estimated in GAEZ v3.0) and actual yields and production from downscaling year 2000 statistics of main food and fiber crops (statistics derived mainly from FAOSTAT and the FAO study AT 2010/30). Here we chose to use GYGA values since it contains more recent data. However, a comparison between GYGA and GAEZ reveals that their results are aligned[8].
- Threshold values based on GYGA:
    - *Satisfactory*: ≤ 30%
    - Need for minor improvements: 30-60%
    - **Need for major improvements**: ≥ 60%

Indicator 2.3.2. "Rural poverty headcount ratio at national poverty lines" (% of rural population)

- Justification: A country's poverty line consists of the minimum level of income deemed adequate in that country. Although countries may calculate it in slightly different ways, the poverty line offers an indication of the level of poverty within a population. "Rural poverty headcount ratio at national poverty lines" corresponds to the percentage of the rural population living below the national poverty lines[9]. This is a useful parameter to assess whether agricultural

---

[6] Available at: http://www.who.int/gho/ncd/risk_factors/overweight/en/.
[7] Agro-ecological Zones (GAEZ) FAO & IIASA - http://www.gaez.iiasa.ac.at/. Further methodological details are available at: http://www.fao.org/nr/gaez/about-data-portal/yield-and-production-gaps/en/#
[8] GAEZ yield gap values obtained for Nigeria, Brazil and the Netherlands were 69%, 49% and 9%, respectively.
[9] Jolliffe, Dean Mitchell; Prydz, Espen Beer. 2016. *Estimating international poverty lines from comparable national thresholds*. Policy Research working paper; no. WPS 7606; Paper is funded by the Knowledge for Change Program (KCP). Washington, D.C.: World Bank Group. Available at:

activities can support livelihoods, complementary to indicator 2.3.3 (on changes in average income levels). To be able to compare different countries' poverty lines, it is important to correct them to the countries' relative purchasing power parity (PPP), that is, their ability to purchase a given basket of goods in their respective currency.

Table S1 - National poverty lines as defined by the World Bank based on data from the World Bank and OECD. "PPP" refers to purchasing power parity. National poverty lines define the daily threshold value considered in our classification.

	Year	National poverty line (2011 PPP $ day^{-1})	Source
Nigeria	2010	1.65	World Bank
Brazil	2011	3.05	World Bank
Netherlands	2012	26.31	OECD

- Calculation of national poverty lines (World Bank): "National poverty lines are estimated by combining national poverty headcounts from national sources, reported in the World Bank's databases, with corresponding consumption and income distributions from PovcalNet used for international poverty estimates. Further, because the consumption and income distributions we use are all expressed in per capita PPP terms, the estimated national poverty lines are all expressed in comparable per capita PPP dollars. (…) This produces a series of poverty lines that are closer to the ICP reference year (thereby reducing the sensitivity of the estimate to errors and updates in inflation data)."
- Source: World Bank, Global Poverty Working Group. Poverty and equity database: http://povertydata.worldbank.org/poverty/home/
- Threshold values proposed:
    - *Satisfactory*: 0% (i.e. entire population above poverty line)
    - Need for minor improvements: ≤ 5%
    - **Need for major improvements**: > 5%

Indicator 2.3.3. "Prevalence of farmers earning less than the national minimum wage" (%)

- Justification: Farmers' income is crucial for food security, as it allows or constrains farmers' access to inputs as well as food products.
- Definition/calculation: National minimum wages are based on the "Statutory nominal gross monthly minimum wage" proposed by the International Labor Organization (ILO). Values are based on a common currency (USD) so that countries can be compared (see table S2). According to ILO, "In cases where a national minimum wage is not mandated, the minimum wage in place in the capital or major city is used. In some cases, an average of multiple regional minimum wages is used. In countries where the minimum wage is set at the sectoral level or occupational level, the minimum wage for manufacturing or unskilled workers is generally applied." The earning of farmers was calculated per country, based on national statistics.

---

http://documents.worldbank.org/curated/en/837051468184454513/Estimating-international-poverty-lines-from-comparable-national-thresholds

Table S2 – National minimum monthly wages in USD, as defined by ILO[10]

	Min. wage (nominal USD)
Nigeria	114 (2013)
Brazil	307 (2014)
Netherlands	1962 (2013)

- Sources: National databases
    - Nigeria: NBS
    - Brazil: IBGE/DIEESE
    - Netherlands: LEI/FADN
- Threshold values proposed:
    - *Satisfactory*: ≤ 5%
    - Need for minor improvements: 5-10%
    - **Need for major improvements**: ≥ 10%

Target 2.4

Indicator 2.4.1. "Water withdrawn by agriculture as a percentage of total water withdrawal" (%)

- Justification: This indicator highlights the pressure on the renewable water resources caused by irrigation.
- Definitions according to the Aquastat metadata: [Agricultural water withdrawal as % of total water withdrawal] = [Agricultural water withdrawal] / [Total water withdrawal] *100.
    - "Agricultural water withdrawal" (10^9 m^3 yr^{-1}): Annual quantity of self-supplied water withdrawn for irrigation, livestock and aquaculture purposes. It can include water from primary renewable and secondary freshwater resources, as well as water from over-abstraction of renewable groundwater or withdrawal from fossil groundwater, direct use of agricultural drainage water, direct use of (treated) wastewater, and desalinated water. Water for the dairy and meat industries and industrial processing of harvested agricultural products is included under industrial water withdrawal.
    - "Total water withdrawal" (10^9 m^3 yr^{-1}): Annual quantity of water withdrawn for agricultural, industrial and municipal purposes. It can include water from primary renewable and secondary freshwater resources, as well as water from over-abstraction of renewable groundwater or withdrawal from fossil groundwater, direct use of agricultural drainage water, direct use of (treated) wastewater, and desalinated water. It does not include in-stream uses, which are characterized by a very low net consumption rate, such as recreation, navigation, hydropower, inland capture fisheries, etc.

---

[10]Available at: http://www.ilo.org/ilostat/faces/oracle/webcenter/portalapp/pagehierarchy/Page27.jspx?subject=EAR&indicator=EAR_4MMN_CUR_NB&datasetCode=A&collectionCode=YI&_afrLoop=180653359024785&_afrWindowMode=0&_afrWindowId=null#!%40%40%3Findicator%3DEAR_4MMN_CUR_NB%26_afrWindowId%3Dnull%26subject%3DEAR%26_afrLoop%3D180653359024785%26datasetCode%3DA%26collectionCode%3DYI%26_afrWindowMode%3D0%26_adf.ctrl-state%3D28s7wfoxx_50

- Source: Aquastat - http://www.fao.org/nr/water/aquastat/data/query/ (variable 4254)
- Threshold values proposed:
    - *Satisfactory*: ≤ 15%
    - Need for minor improvements: 15-40%
    - **Need for major improvements**: ≥ 40%

Indicator 2.4.2. “Average water productivity in agriculture” (kg m^{-3} yr^{-1})

- Definition according to the Aquastat metadata: Annual quantity of self-supplied water withdrawn for irrigation, livestock and aquaculture purposes. It can include water from primary renewable and secondary freshwater resources, as well as water from over-abstraction of renewable groundwater or withdrawal from fossil groundwater, direct use of agricultural drainage water, direct use of (treated) wastewater, and desalinated water. Water for the dairy and meat industries and industrial processing of harvested agricultural products is included under industrial water withdrawal.
- Note: This indicator offers an aggregate measure. It could be that some countries produce more water-intensive crops than others.
- Calculation: Total crop production (megatons) / Total agricultural water withdrawal ($10^9 m^3$ yr^{-1})
    - Total crop production - FAOSTAT Database - http://www.fao.org/faostat/en/#data
    - Total agricultural water withdrawal - Aquastat Database >> Water withdrawal by sector (variable 4250) - http://www.fao.org/nr/water/aquastat/water_use/index.stm
- Source: Indicated above
- Threshold values proposed:
    - *Satisfactory*: ≥ 500 kg m^{-3} yr^{-1}
    - Need for minor improvements: 200-500 kg m^{-3} yr^{-1}
    - **Need for major improvements**: ≤ 200 kg m^{-3} yr^{-1}
- Note: Water productivity, as measured here, is not independent from the availability and productivity of other inputs. Yet, indicator 2.4.2 focuses on the efficiency with which water resources are used, irrespective of the share of yield losses directly attributable to water limitation or to its potential interaction with other factors.

Indicator 2.4.3. “Nitrogen use efficiency” (kg N kgN^{-1})

- Justification: Improvements in nitrogen use efficiency (NUE) in crop production are critical for food security, environmental degradation and climate change.
- Definition (Zhang et al. 2015): Nitrogen Use Efficiency (kg N kgN^{-1}) is the ratio of outputs (i.e. crop output) to inputs (fertilizer, biological N fixation, manure, deposition). In short: NUE $= N_{yield}/N_{input}$
- Source: Zhang et al. 2015 Nature (Online Supplementary Material) http://www.nature.com/nature/journal/v528/n7580/abs/nature15743.html#supplementary-information

- Threshold values based on the EU Nitrogen Expert Panel (2015)[11]:
  - *Satisfactory*: 0.5-0.9 (desired range)
  - Need for minor improvements: 0.4-0.5 or 0.9-1.0 (close to desired range)
  - **Need for major improvements**: ≤ 0.4 (rationale: lower levels exacerbate N pollution) or ≥ 1.0 (rationale: higher values risk mining of soil Nitrogen stock)
- Notes:
  - Some global nitrogen figures presented by Zhang et al. (2015) largely deviate from national estimates in the Netherlands. According to Zhang et al. (2015), in 2011, NUE (indicator 2.4.3) was shown to be 0.26 kg N kgN^{-1} and nitrogen surplus (indicator 2.4.4) was shown to be 369 kg N km^{-2}. These numbers were based on unrealistic nitrogen application rates (e.g. 620 kg N ha^{-1} for wheat) which, together with realistic N harvested rates (e.g. 152 kg N ha^{-1} for wheat), leads to excessively high N surplus and low NUE. According to other scientific publications and official national statistics[12], the Dutch NUE was estimated to be almost twice as high and the and nitrogen surplus of less than half.
  - Notwithstanding the importance of global estimates in eliciting regional differences and country priorities, apparent inconsistencies reinforce the need to cross-check global and national values whenever the latter is available.

Indicator 2.4.4. "Average nitrogen surplus" (ton N km^{-2})

- Justification: Nitrogen balance indicates the potential surplus of nitrogen on agricultural land and the associated pressure on soil, air and water.
- Definition (Zhang et al. 2015): Av. nitrogen surplus (N_{sur}) is the difference between inputs (i.e. fertilizer, agricultural biological fixation, manure and deposition) and outputs (i.e. N content in the crop product), which corresponds to the sum of N losses to the environment (either through volatilization, leaching or run-off) plus N recycling within the soil. In short: $N_{sur} = N_{input} - N_{yield}$
- Source: Zhang et al. 2015 Nature (Online Supplementary Material) http://www.nature.com/nature/journal/v528/n7580/abs/nature15743.html#supplementary-information
- Threshold values based on the EU Nitrogen Expert Panel (2015):
  - *Satisfactory*: ≤ 3.9 ton N km^{-2} (desired range)
  - Need for minor improvements: 4.0-8.0 ton N km^{-2} (close to desired range)
  - **Need for major improvements**: ≥ 8.0 ton N km^{-2} (attention required)
- Notes: Same as above (indicator 2.4.3).

---

[11] EU Nitrogen Expert Panel (2015) *Nitrogen Use Efficiency (NUE) - an indicator for the utilization of nitrogen in agriculture and food systems*. Wageningen University, Alterra, PO Box 47, NL-6700 Wageningen, Netherlands. Available at www.eunep.com

[12] For example, see:

- Lassaletta, L., Billen, G., Grizzetti, B., Anglade, J. and Garnier, J., 2014. 50-year trends in nitrogen use efficiency of world cropping systems: the relationship between yield and nitrogen input to cropland. *Environmental Research Letters*, *9*(10), p.105011.
- Prins, 2017 – WUR/LEI (http://edepot.wur.nl/407050).
- PBL, 2017. Evaluatie Meststoffenwet 2016: Syntheserapport (n. 2258). Den Haag.
- CBS, 2018. Duurzame ontwikkelingsdoelen: de stand voor Nederland. Den Haag.

Indicator 2.4.5. “GHG emission intensity of food production” (Mg CO_2e M $kcal^{-1}$)

- Justification: Agriculture accounts for nearly a third of global GHG emissions (IPCC). Agriculture-related GHG emissions are expected to increase in the future pushed by demand growth, posing further threats to the world’s food and climate security. Efforts are needed to reduce GHG emissions coming from the agricultural sector. Indicator 2.4.5 measures the GHG emission intensity of agriculture per country, that is, the level of GHG emissions per food kcal produced.
- Calculation: “2000-era emissions from a 200-iteration Monte Carlo simulation include CH_4 emissions from rice paddies, CO_2, N_2O, and CH_4 emissions from peatland drainage, and N_2O emissions from manure and synthetic N application. Production intensity includes all crop calories. Food intensity excludes calories dedicated to industrial and non-food uses [using the FAOSTAT Food Balance Sheets] and assumes that 12% of the calories used as livestock feed are available in foods for human consumption (Cassidy et al. 2013).” Also, “using bilateral crop trade data[13], the authors linked imported crop use to producing-country calorie production.”
- Source: Carlson et al., 2015 – Nature Climate Change (Online Supplementary Material 2) https://www.nature.com/nclimate/journal/v7/n1/full/nclimate3158.html#supplementary-information
- World distribution (236 countries, mean* = 0.19, std. dev. = 0.81):

Cum. Freq.	Cum.%	Value
177	75.00	≤ 0.14
192	81.36	0.14-0.19*
236	100.00	≥ 0.19

- Threshold values based on the world statistical distribution (above):
  - *Satisfactory*: ≤ 0.14
  - Need for minor improvements: 0.14-0.19
  - **Need for major improvements**: ≥ 0.19

Indicator 2.4.6. “Average carbon content in the topsoil” (% in weight)

- Justification: Several attempts have been made to identify appropriate indicators of soil quality and land degradation, and to measure them worldwide (Gibbs & Salmon 2015). Soil organic carbon has been considered by EUROSTAT an appropriate indicator of soil quality, as high organic carbon content usually corresponds to good agro-environmental conditions.
- Notes:
  - It is challenging to define a single value for average carbon content in the topsoil at the country level. Although the FAO adopts it as a soil quality indicator, it is necessary to complement country-level indicators with finer data at the subnational level.
  - The UNEP-funded GLASOD project (http://www.isric.org/projects/global-assessment-human-induced-soil-degradation-glasod) has produced a world map of human-induced soil degradation using an expert-based approach. The results of this project were

[13] MacDonald, G.K., Brauman, K.A., Sun, S., Carlson, K.M., Cassidy, E.S., Gerber, J.S. and West, P.C., 2015. Rethinking agricultural trade relationships in an era of globalization. *BioScience*, *65*(3), 275-289.

    generated in 1991 and 1998. Although they still feature in the FAOSTAT database as soil quality indicators, a more up-to-date measure is preferred.
  - Better statistics on soil degradation (based on multiple parameters related to the various functions of the soil) will be made available in the future through the new version of the FAO GLADIS dataset.
- Calculation: The data used to generate this indicator are geo-spatial raster data contained in the Harmonized World Soil Database (HWSD) released by FAO, IIASA, ISRIC, ISSCAS, and JRC in 2008 with a spatial resolution of 30 by 30 arc seconds (approximately 1 Km). Spatial data were extracted through appropriate queries from the geo-database and then spatial statistics were calculated at the country level.
- Source: FAOSTAT Database (Item code: 6709, Element code: 7221) http://www.fao.org/faostat/en/#data/ES
- Threshold values based in the soils with organic carbon content (% in weight):
  - *Satisfactory*: ≥ 2% (soils with high agricultural value).
  - Need for minor improvements: 1-2% (soils which are apt for agriculture but have limited agricultural value)
  - **Need for major improvements**: ≤ 1% (soils which are generally affected by degradation processes and erosion)

Indicator 2.4.7. "Climate change vulnerability index for food" [0-1]

- Justification: Vulnerability measures the exposure, sensitivity and adaptive capacity towards the negative impacts of climate change. This indicator is based on crucial elements for the achievement of food security and agricultural sustainability.
- Definition: The vulnerability index for food is composed of 6 elements:
  - projected change of cereal yield, incl. rice, wheat and maize (*2040-2069 under RCP4.5 according to results from EPIC, GEPIC, LPJmL, pDSSAT, PEGASUS*);
  - projected population change (*2020-2050*);
  - food import dependency (*1995-2014 – FAOSTAT*);
  - rural population as a share of total population (*1995-2014 – WDI*);
  - capacity to acquire and deploy agriculture technology (e.g. amount of fertilizer use, amount of pesticide use, ability to equip agriculture area with irrigation, the frequency of tractor use and child malnutrition) (*various*).
- Source: ND-GAIN Country Vulnerability Index for food - http://index.gain.org/
- Threshold values based on the world distribution percentiles:
  - *Satisfactory*: ≤ 0.426 (highest third, i.e. 64 countries with highest scores)
  - Need for minor improvements: 0.426 - 0.627 (middle third)
  - **Need for minor improvements**: ≥ 0.627 (lowest third, i.e. 64 countries with lowest scores)

Indicator 2.4.8. "Use of pesticides per area" (kg ha^{-1})

- Justification: Pesticides pose a major threat to genetic diversity in agriculture. Its continued and abusive use may lead to the oversimplification of agroecosystems. The proposition of an

indicator related to the use of pesticides per area serves as an incentive to the application of alternative pest control techniques, such as integrated pest management, employment of pest prevention measures, etc.

- Calculation: This indicator includes data on the use of major pesticide groups (insecticides, herbicides, fungicides) and of relevant chemical families. It is measured in tons of active ingredients used in the agricultural sector for crops and seeds.
- Notes:
    - A weighted average of the use of pesticides and their associated level of toxicity cannot be calculated at the global level due to data limitations. However, it should be considered at the national level, whenever possible.
    - Values for Nigeria are unavailable, but the indicator was kept due to its broader geographic coverage when compared to alternative indicators on pesticides.
- Source: FAOSTAT Database (element code 5161) - http://www.fao.org/faostat/en/#data/RP
- Threshold values proposed:
    - *Satisfactory*: ≤ 5 kg ha^{-1}
    - Need for minor improvements: 5-10 kg ha^{-1}
    - **Need for major improvements**: ≥ 10 kg ha^{-1}

Target 2.5

Indicator 2.5.1. "Average number of gaps in *ex-situ* collections of selected crop genepools"

- Justification: Indicator 2.5.1 is related to genetic resources for food and agriculture secured in conservation facilities. Data on the complete collection of seedbanks and germplasm banks (incl. which species are secured and what is their country of origin) is limited. However, gaps in the taxonomic and geographic coverage of Crop Wild Relatives[14] in *ex-situ* collections are known and offer a good proxy. A recent study by CIAT and Bioversity International has highlighted these gaps for a number of genepools. The indicator we propose relies on the number of gaps found amongst 12 major crops produced and consumed worldwide, given where each species is expected to occur for these crops based on herbarium specimens.
- Calculation: The scores assigned to each country are based on the predominant values shown in figure 1.2 of the FAO report "The Second Report on the State of the World's Plant Genetic Resources for Food and Agriculture" (http://www.fao.org/docrep/013/i1500e/i1500e.pdf).
- Source: http://gisweb.ciat.cgiar.org/gapanalysis/
- Threshold values proposed:
    - *Satisfactory*: ≤ 1
    - Need for minor improvements: 1-3
    - **Need for major improvements**: ≥ 3

[14] Crop wild relatives are wild plant species that are genetically related to cultivated crops. Untended by humans, they continue to evolve in the wild, developing traits – such as drought tolerance or pest resistance – that farmers and breeders can cross with domesticated crops to produce new varieties.

Indicator 2.5.2. “Proportion of local breeds classified as being at risk out of all breeds whose risk of extinction is known” (%)

- Justification: The risk status of major species offers an indication of the conservation of genetic resources in each country and the need for further action.
- Notes:
    - This indicator is measured relative to the number of species whose risk of extinction is known as a way of reducing distortions among countries with more or less catalogued species. Efforts aimed at species monitoring is a component of SDG 15 (“Life on land”).
    - It seems desirable that the proportion of types at risk is as low as possible. However, the more diverse a system is, the more breeds will be relatively rare and therefore more vulnerable to extinction. Likewise, a system with only one or a few abundant breeds has 0% breeds at risk of extinction, but very low diversity. Once again, this underscores the importance of implementing the SDG Agenda in its integrity; SDG 15 deals with biodiversity as a whole and is complementary to indicator 2.5.2.
- Source: FAO report "Status and trends of animal genetic resources", 2016 (Annex 2)
- Threshold values proposed:
    - *Satisfactory*: ≤ 10 %
    - <u>Need for minor improvements</u>: 10-20 %
    - **Need for minor improvements**: ≥ 20 %

Historical trends for specific indicators

Target 2.1.

a

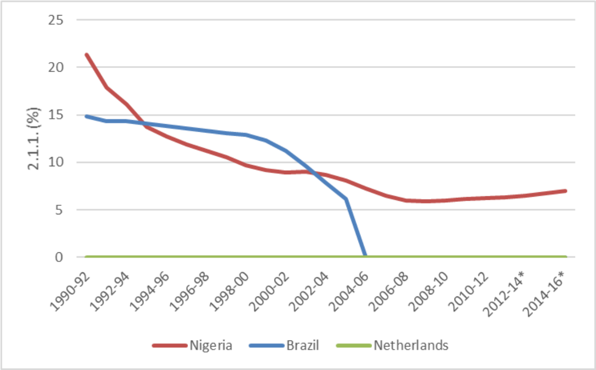

b

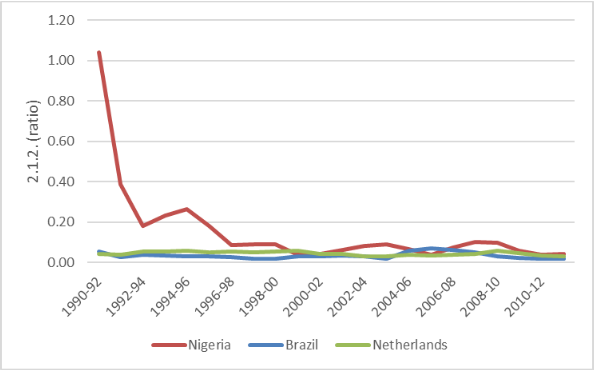

c

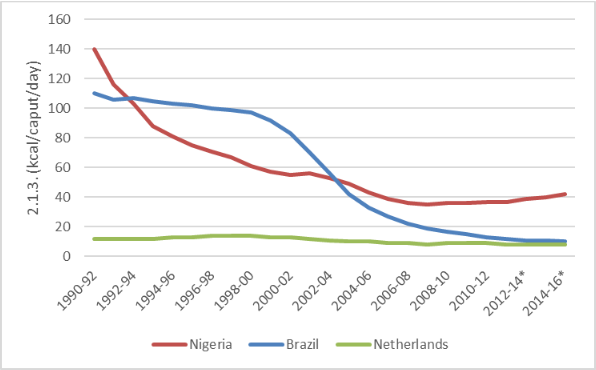

*Figure S1 – Historical trends of Nigeria, Brazil and the Netherlands with regards to indicators **(a)** 2.1.1. "Prevalence of undernourishment"; **(b)** 2.1.2. "Per capita food supply variability index"; and **(c)** 2.1.3. "Depth of the food deficit".*

Target 2.2.

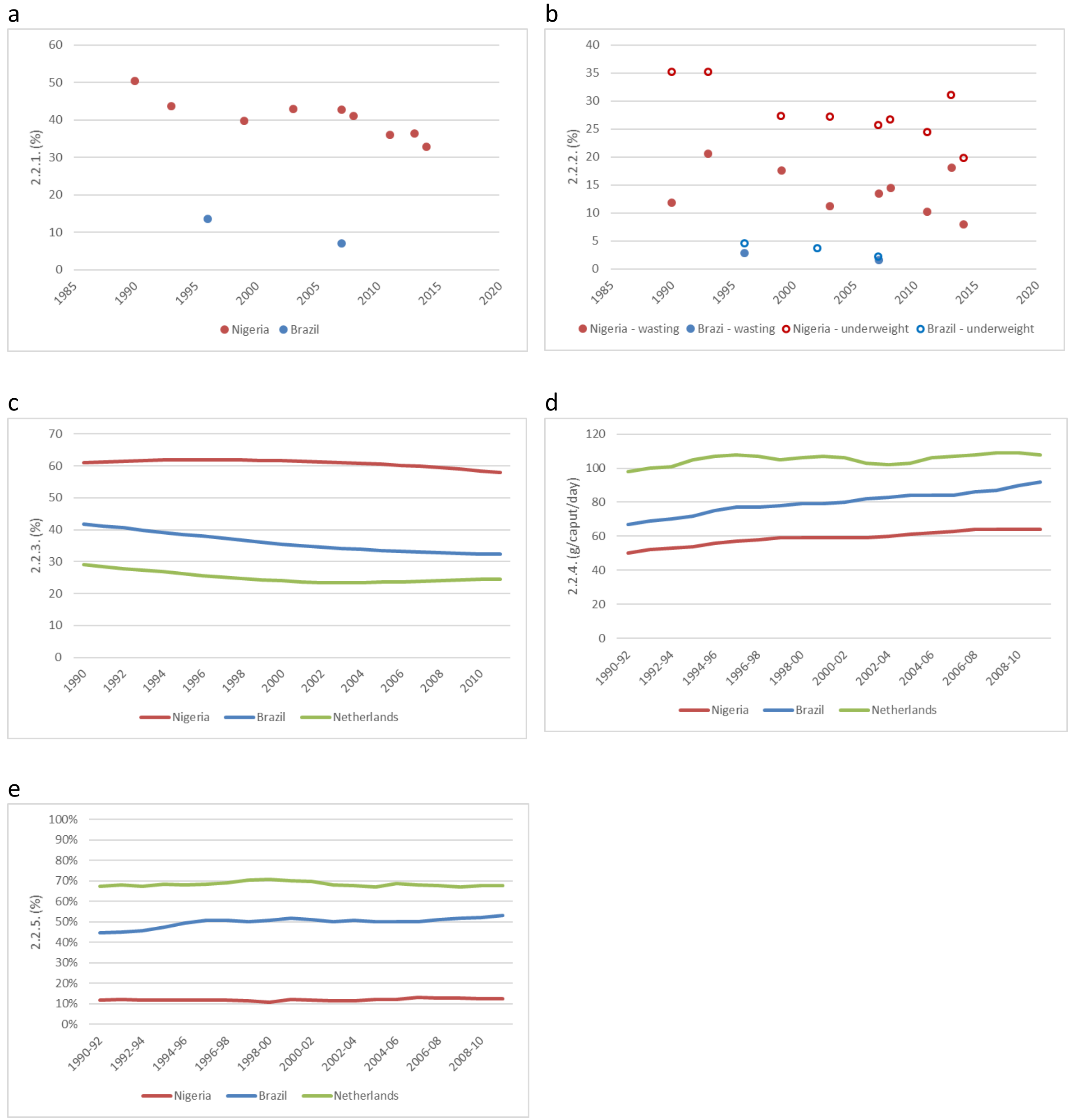

*Figure S2 – Historical trends of Nigeria, Brazil and Netherlands with regards to indicators **(a)** 2.2.1. "Prevalence of stunting among children under 5 years of age"; **(b)** 2.2.2. "Prevalence of malnutrition among children under 5, disaggregated by wasting and underweight"; **(c)** 2.2.3. "Prevalence of anemia among pregnant women"; **(d)** 2.2.4. "Average protein supply"; and **(e)** 2.2.5. "Share of average protein supply of animal origin". Data on obesity levels are not available for previous years.*
